# Successful identification of culprit drugs of perioperative anaphylaxis by repeated skin testing after negative first skin tests in a patient with a long distant history of perioperative anaphylaxis

**DOI:** 10.1016/j.heliyon.2021.e08401

**Published:** 2021-11-16

**Authors:** Wasurat Sungworn, Orathai Theankeaw, Aree Jameekornrak Taweechue, Chamard Wongsa, Torpong Thongngarm, Mongkhon Sompornrattanaphan

**Affiliations:** a Adverse Drug Reaction (ADR) Unit, Pharmacy Department, Siriraj Hospital, Mahidol University, Bangkok, Thailand; b Division of Allergy and Clinical Immunology, Department of Medicine, Faculty of Medicine Siriraj Hospital, Mahidol University, Bangkok, Thailand

**Keywords:** Anaphylaxis, Anesthesia, Hypersensitivity reactions, Intraoperative, Perioperative period

## Abstract

**Background:**

Perioperative anaphylaxis is a severe immediate hypersensitivity reaction to drugs administered in immediate temporal association to surgical procedures. The European Academy of Allergy and Clinical Immunology recommends allergologic tests be performed within the golden period of between 1 and 4 months after the date of the event to avoid false negatives. Nonetheless, many obstacles prevent patients from receiving diagnostic tests within the recommended time frame.

**Case presentation:**

A 39-year-old male with congenital glaucoma had a history of multiple episodes of perioperative anaphylaxis since the age of 1 year including generalized urticaria, bronchospasm, cyanosis, and hypotension. Because the sequence of events was unclear due to incomplete documentation of operations and the destruction of medical records, the allergists tested different perioperative drugs on the patient. Although the first test results were all negative, repeated tests at 6 weeks were positive for morphine and ketamine. We identified more than one causative drug at the second round of skin tests. Using recommended skin test concentrations, negative skin tests in 5 control subjects could support the validity of the second test. The patient underwent sinus surgery in the next 3 months after the second skin test using propofol, midazolam, sevoflurane, chlorhexidine, and cefazolin without any anaphylactic reactions.

**Conclusions:**

Repeated skin tests after negative results of the first tests may identify the causative drugs, thus providing optimal patient safety, and should be considered under the physician's discretion together with consideration of the severity of the allergic symptoms, time interval from last reactions, and the patient's consent.

## 1 Introduction

Anaphylaxis is defined as an acute, potentially life-threatening systemic reaction with a wide range of clinical manifestations [1]. Perioperative anaphylaxis is the anaphylaxis associated with anesthesia. The reported incidence has ranged from 1:353–1:18600 procedures from the United Kingdom Sixth National Audit Project (NAP6) [2], 1:3180 from a French prospective study [3], and 1:1480 from a Spanish prospective study [4]. This condition is challenging as many drugs are administered simultaneously, resulting in difficulty to conclude the culprit drugs. Therefore, allergologic workups, including *in vitro* and *in vivo* tests, are important to identify culprit drugs and safe alternatives [5, 6].

Both European and American guidelines currently recommend using skin tests as the main diagnostic method for evaluating drug hypersensitivity reactions caused by any general medication [7]. The European Academy of Allergy and Clinical Immunology (EAACI) recommends allergologic tests be performed within the golden period of between 1 and 4 months after the date of the anaphylactic event to avoid false negatives [5, 8]. Nonetheless, many obstacles prevent patients from receiving diagnostic tests within the recommended time frame. Herein, we report a patient with a history of perioperative anaphylaxis 10 years prior to allergologic evaluation. There were two rounds of test, for which the second round was 6 weeks after the first. The first round of skin tests showed negative results, but the second round of tests repeated using the same drugs as the first were positive for ketamine and morphine, which were identified as the culprit drugs.

## 2 Case presentation

A 39-year-old male with congenital glaucoma, epilepsy, hypertension, dyslipidemia, and diabetes and without a history of atopic disease had a history of multiple eyes examinations under anesthesia since the age of 1 year. Nonetheless, most of the medical records were narrative notes, and some of the anesthetic and medication records could not be retrieved. As a result, the medication list might have been incomplete.

The patient's history relating to allergy is summarized in Table 1**.** He developed his first episode of anaphylaxis at the age of 11 years during trabeculectomy performed under general anesthesia (GA) with thiopental and succinylcholine at a local hospital. The event was documented as a shock of unknown etiology. The second and third episodes of anaphylaxis occurred at the ages of 13 and 14 years, both of which involved cyanosis and hypotension shortly after induction of anesthesia during the eye examination. Consequently, the local hospital referred the patient to an academic hospital and issued an adverse drug reaction (ADR) card stating a suspicion of thiopental and succinylcholine allergy by guessing.

**Table 1:** Summary of the patient's allergic history prior to the most recent set of visits involving skin tests.

Anaphylaxis episode	Age (years)	Drugs used during operation	Reactions
1st	11	(At least) thiopental and succinylcholine	Bronchospasm, cyanosis, and hypotension
2nd	13	(At least) thiopental and succinylcholine	Bronchospasm, cyanosis, and hypotension
3rd	14	Premedication: chlorpheniramine, dexamethasone (At least) thiopental, succinylcholine, atracurium **ADR card: avoid thiopental and succinylcholine**	Bronchospasm, prolonged cyanosis, and hypotension
Multiple episodes	15–28	Avoid thiopental and succinylcholine. Unknown definite medication used during operation **ADR card was revised to: avoid thiopental and morphine**	3 episodes of anaphylaxis
Last episode	29	Enucleation operation: unknown medications used **ADR card was revised to: avoid all perioperative drugs except for propofol, succinylcholine, midazolam, sevoflurane**	Generalized urticaria, bronchospasm, and hypotension

Abbreviation: ADR, adverse drug reaction.

During the ages of 15–28 years, he had multiple surgical operations at the same academic hospital to which he had been referred. All operative and anesthetic records had been previously destroyed due to the prolonged loss of contact for more than five years. The patient claimed the hospital did not use thiopental and succinylcholine according to the ADR card registered by the first local hospital. Nevertheless, the patient still suffered from three more episodes of anaphylaxis. Therefore, in accordance with the ADR unit, an anesthesiologist at the academic hospital specified the patient could be allergic to morphine and thiopental in the ADR card using informed guesswork. Nevertheless, the patient had an eye enucleation operation under GA without morphine and thiopental at the age of 29 years and developed anaphylaxis. After that event, the patient and his relatives were very frightened and discouraged, and they refrained from coming to the hospital for 10 years. The latest ADR record described an allergy to all anesthetic drugs except for propofol, succinylcholine, midazolam, and sevoflurane.

Ten years later, the patient visited our university hospital with chronic nasal congestion and was consequently diagnosed with chronic rhinosinusitis (CRS). He had no peripheral eosinophilia (absolute eosinophils = 201 cells/μL). He was treated with a 14-day course of amoxicillin/clavulanic due to recurrent bacterial sinusitis without any reactions. The otolaryngologist planned to perform an operation, so he referred the patient to an allergist to evaluate drug allergy. He was tested with different perioperative drugs according to Table 2**.** Standard concentrations according to the EAACI recommendation were used except for morphine, for which a lower than the recommended concentration [5] was used because our experience suggests morphine at the recommended concentration usually provides a high false-positive rate in Thai patients. A skin prick test (SPT) was performed first on the forearm with the negative and positive control using normal saline and 10 mg/mL of histamine, respectively. Results were considered positive if a wheal diameter of ≥3 mm was read at 20 min. When the SPT was negative, we performed an intradermal test (IDT) on the volar side of the forearm. Results were considered positive if the wheal increased in diameter ≥3 mm compared to the original wheal with concurrent flare.

**Table 2:** Summary of skin tests results.

Drug name	Concentration	Skin prick test		Concentration	Intradermal test	
		1st test	2nd test		1st test	2nd test
Negative control (NSS)	0.9% NaCl	neg	neg	0.9% NaCl	neg	neg
Positive control (Histamine)	10 mg/mL	5 × 5 mm	6 × 5 mm	-	N/A	N/A
Succinylcholine (Suxamethonium®)	10 mg/mL	neg	neg	0.1 mg/mL	neg	neg
Atracurium (Tracrium®)	1 mg/mL	neg	neg	0.01 mg/mL	neg	neg
Cisatracurium (Nimbex®)	2 mg/mL	neg	neg	0.02 mg/mL	neg	neg
Rocuronium (Esmeron®)	10 mg/mL	neg	neg	0.05 mg/mL	neg	neg
Etomidate (Lipuro®)	2 mg/mL	neg	neg	0.2 mg/mL	neg	neg
Ketamine	100 mg/mL	neg	neg	0.1 mg/mL	neg	pos^a^
Propofol	10 mg/mL	neg	neg	1 mg/mL	neg	neg
Thiopental	25 mg/mL	neg	neg	2.5 mg/mL	neg	neg
Morphine	1 mg/mL	neg	neg	0.005 mg/mL	pos^b^	pos^c^
Fentanyl	0.05 mg/mL	neg	neg	0.005 mg/mL	neg	neg
Midazolam	5 mg/mL	neg	neg	0.05 mg/mL	neg	neg
Chlorhexidine	5 mg/mL	neg	neg	0.002 mg/mL	neg	neg

**Abbreviations:** neg, negative skin test; pos, positive skin test; EAACI, European Academic of Allergy and Clinical Immunology; N/A, not applicable; NSS, normal saline solution; SPT, skin prick test; IDT, intradermal test.

Notes:-

a **Positive IDT to ketamine:** 3 × 3 mm to 7 × 7 mm (flare 32 × 32 mm).

b **Positive SPT to morphine**: 4 × 3 mm (flare 12 × 10 mm).

c **Positive IDT to morphine** (0.005 mg/mL) 3 × 3 mm → 8 × 8 mm (flare 29 × 27 mm), **IDT to morphine** (0.001 mg/mL) 3 × 3 mm → 6 × 6 mm (flare 18 × 15 mm).

**Figure 1:**
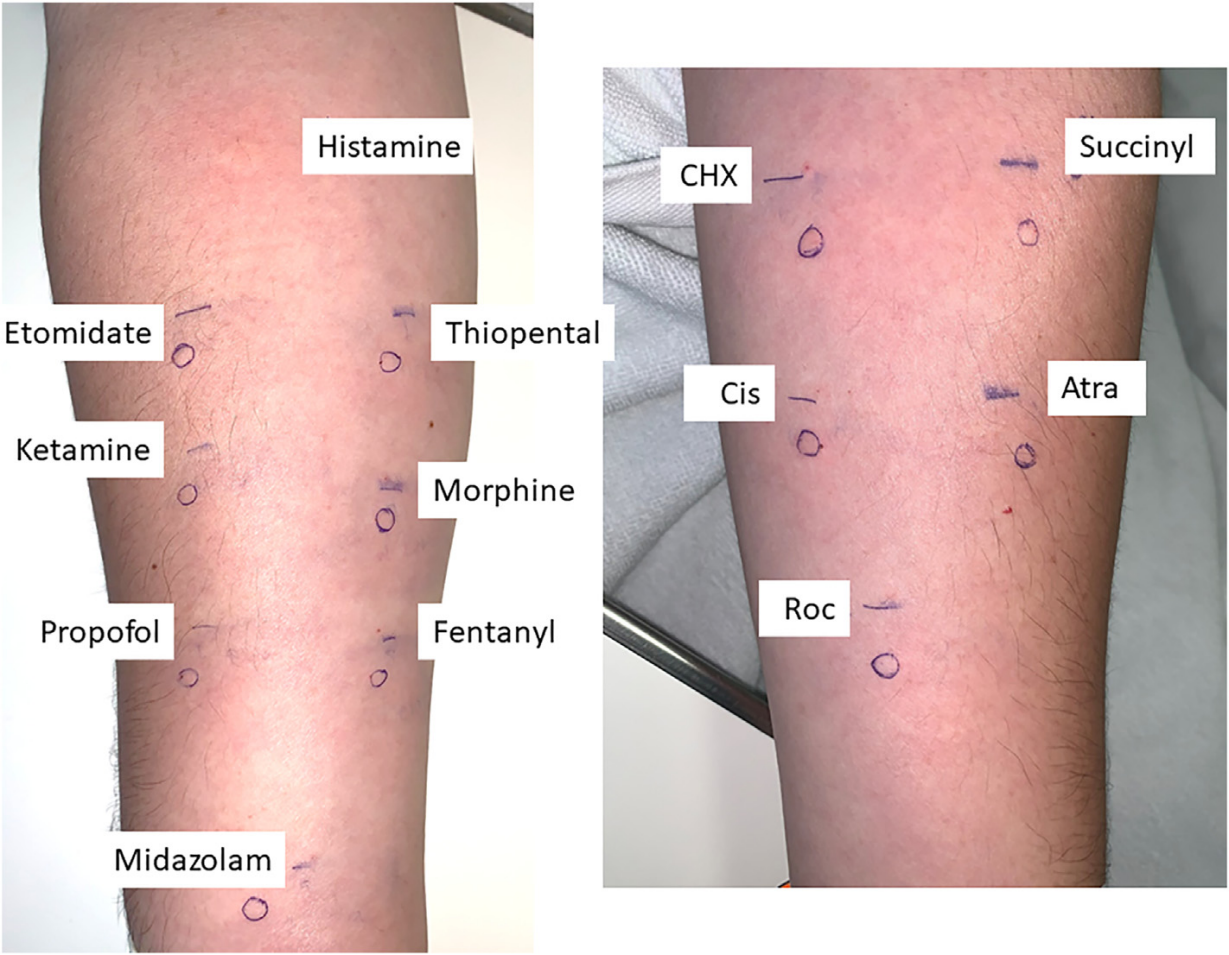
The first skin test results were all negative. **Abbreviations**: Atra, atracurium; CHX, chlorhexidine; Cis, cis-atracurium; Roc, rocuronium; Succinyl. succinylcholine.

**Figure 2:**
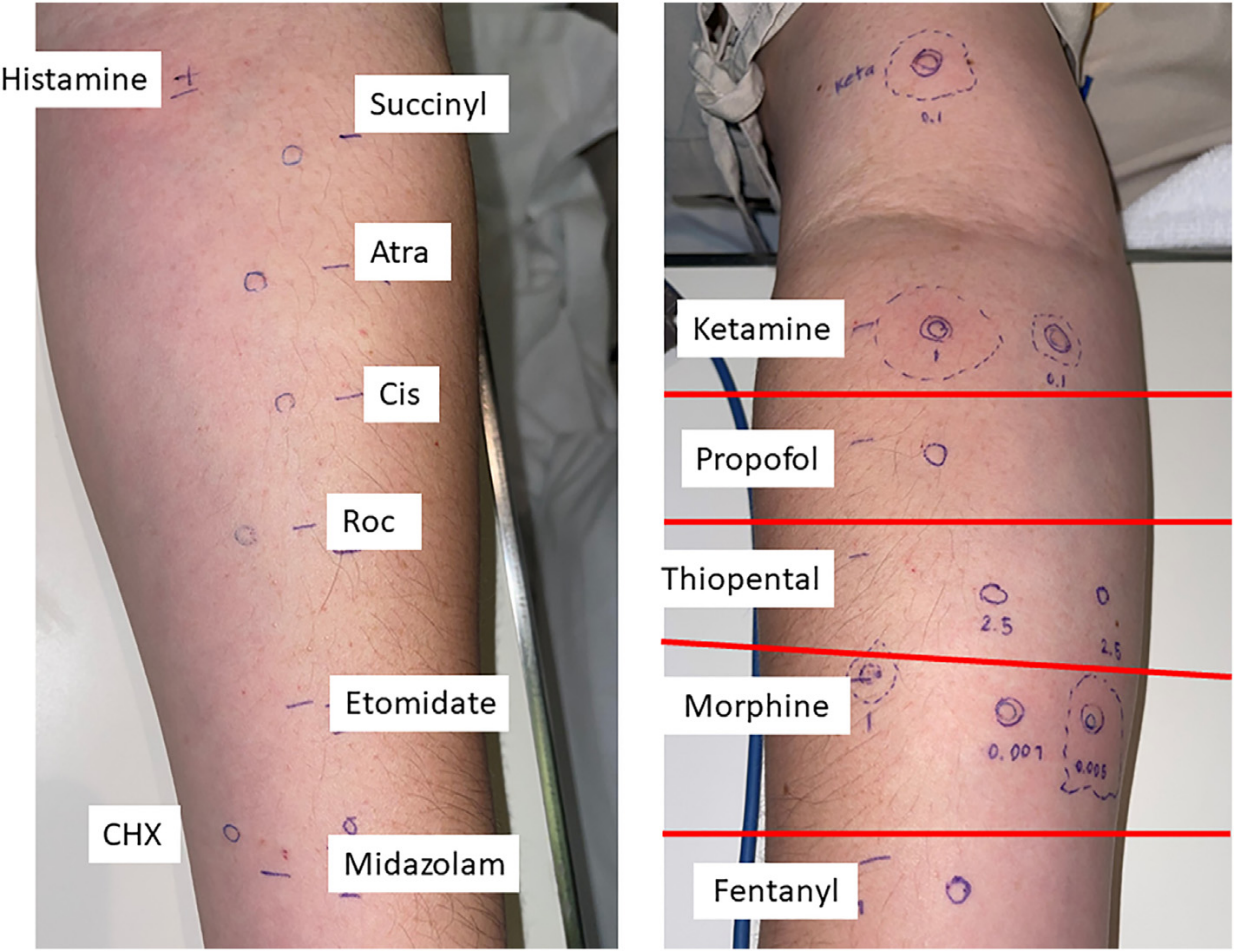
The second skin test results at 6 weeks were positive for ketamine by intradermal test and morphine by skin prick test. **Abbreviations**: Atra, atracurium; CHX, chlorhexidine; Cis, cisatracurium; Roc, rocuronium; Succinyl, succinylcholine.

The first test results were negative. The patient's baseline serum tryptase was 3.82 μg/L, latex-specific immunoglobulin E (IgE) was 0 KUA/L, and complement 4 (C4) levels were 20 and 24 mg/dL at 2 different timepoints (normal range, 15–45 mg/dL). Because the patient had had severe immediate reactions, and all the initial test results were negative, we scheduled the next round of testing 6 weeks later. The second round of skin tests was performed with the same drugs at the same concentrations as the first tests. IDTs were positive for ketamine at 1 and 0.1 mg/mL concentrations, and SPTs were positive for morphine at 1 mg/mL concentration (Table 2). Positive tests for morphine were confirmed with SPTs performed at another two skin sites and negative skin tests to morphine and ketamine in healthy controls, the patient's brother, and mother (Figures 1 and 2).

We recommended the patients avoid ketamine and morphine. Alternative drugs were those testing negative, including fentanyl, propofol, thiopental, etomidate, midazolam, succinylcholine, cisatracurium, atracurium, and rocuronium. The ADR card was updated accordingly. Nonetheless, close observation for allergic symptoms was advised in the case of future anesthetic procedures with these alternative drugs. The patient underwent sinus surgery in the next 3 months after the second skin test using propofol, midazolam, sevoflurane, chlorhexidine, and cefazolin without any anaphylactic reactions. For all of the case history, images, and data to be published, written and informed consent was obtained from the patient. The patient was informed that de-identified data would be used in the scientific research and publications.

## 3 Discussion and conclusion

To the best of our knowledge, this is the first case report to demonstrate the clinical benefit of repeated skin tests in a patient with a history of severe immediate allergic reactions to perioperative drugs. The utility of the repeated tests in yielding identifications of causative drugs was still evident even after 10 years had passed since the last anaphylaxis. Although the first test results were negative, the repeated tests at 6 weeks were positive for morphine and ketamine, confirmed by the negative skin test of the mentioned drugs in healthy controls. Furthermore, we identified more than one causative drug at the second round of skin tests. The identification of ketamine as a causative drug could be explained the patient's history of anaphylaxis episodes after morphine had been avoided. The rationale of the positive conversion is most likely due to the ‘booster effect’ after the drug re-exposure from the first skin test, resulting in a positive second skin test. Although the patient was exposed the culprit that was eventually identified several times, the lack of drug exposure for a long period could have resulted in a decrease of the drug-specific IgE below the threshold of skin test detection while drug-specific memory B cell may have still been present. This phenomenon has been reported in other drugs [9]. The waning of drug-specific IgE over time could explain the lower detection rate in cases with a long time interval between the last reaction to skin test [10, 11]. We are aware of the possibility of nonspecific irritation from both morphine and ketamine. Using recommended skin test concentrations [5, 12], negative skin tests in the control subjects in the present report could support the validity of the second test.

The 2019 EAACI guideline [5, 12] suggests a repeated test for drug-specific IgE levels at 4–6 weeks after negative initial testing, which is similar to other recommendations [13, 14, 15]. This suggestion is supported by reports on Hymenopteran envenomation and some drugs. A refractory period of up to 6 weeks was reported after Hymenopteran envenomation, and specific IgE might be depleted in the anaphylaxis if the tests are performed too early [16, 17]. Conversely, the waning of drug-specific IgE over time could explain the lower detection rate in cases with a long time interval between the last reaction to the skin test. Opstrup, et al. [18] suggested the optimal sampling time seems to be >1 month and <4 months because they found chlorhexidine-specific IgE declined over months/years. Similar phenomena were demonstrated in antibiotics [10] and iodinated radiocontrast [11]. These findings are supported by a multi-centered European radiocontrast study in which positive test results were more frequent in patients tested within 2–6 months than those tested earlier than 2 months or later than 6 months [11].

However, there has been no evidence that such repeated tests have any utility and clinical benefits before the present report, in which the final outcome was no perioperative drug reaction. Not only could a repeated testing procedure identify the causative drugs, but it could also prevent future severe reactions. Previous studies have found patients with negative first test results could develop an anaphylactic reaction during procedures, implying that their prior test results were false negatives [19, 20]. Moreover, there has been a report of fatal anaphylaxis from a second amoxicillin/clavulanic acid provocation after a prior negative provocation [21].

In the present report, allergists decided to retest and consequently discovered the culprit drugs, which were ketamine and morphine. On the other hand, if only the first test was performed, a recurrent anaphylaxis might have occurred had the patient gone on to surgery without the repeated tests. This would potentially have occurred because one of the identified culprit drugs, ketamine, would not yet have been identified. Therefore, this case report illustrates the substantial benefit of repeated skin tests to prevent future severe reactions and provide optimal patient safety.

In conclusion, repeated skin tests after negative results of the first tests may identify the causative drugs, providing optimal patient safety, and should be considered under the physician's discretion together with consideration of the severity of the allergic symptoms, the time interval from last reactions, and the patient's consent.

## Declarations

### Author contribution statement

All authors listed have significantly contributed to the investigation, development and writing of this article.

### Funding statement

This research did not receive any specific grant from funding agencies in the public, commercial, or not-for-profit sectors.

### Data availability statement

Data will be made available on request.

### Declaration of interests statement

The authors declare no conflict of interest.

### Additional information

No additional information is available for this paper.
